# Neurotransmitter receptors in the life and death of oligodendrocytes

**DOI:** 10.1016/j.neuroscience.2006.08.070

**Published:** 2007-04-14

**Authors:** R. Káradóttir, D. Attwell

**Affiliations:** Department of Physiology, University College London, Gower Street, London WC1E 6BT, UK

**Keywords:** glutamate, NMDA, development, ischemia, cerebral palsy, multiple sclerosis, E_Cl_, chloride reversal potential, mGluR, metabotropic glutamate receptor, NMDA, *N*-methyl-d-aspartate

## Abstract

Oligodendrocytes are crucial to the function of the mammalian brain: they increase the action potential conduction speed for a given axon diameter and thus facilitate the rapid flow of information between different brain areas. The proliferation and differentiation of developing oligodendrocytes, and their myelination of axons, are partly controlled by neurotransmitters. In addition, in models of conditions like stroke, periventricular leukomalacia leading to cerebral palsy, spinal cord injury and multiple sclerosis, oligodendrocytes are damaged by glutamate and, contrary to dogma, it has recently been discovered that this damage is mediated in part by *N*-methyl-d-aspartate receptors. Mutations in oligodendrocyte neurotransmitter receptors or their interacting proteins may cause defects in CNS function. Here we review the roles of neurotransmitter receptors in the normal function, and malfunction in pathological conditions, of oligodendrocytes.

For normal brain function, it is essential that signals pass rapidly between neurons. Oligodendrocytes play an important role in assuring fast neuronal signaling in the CNS. By covering neuronal axons with myelin, which decreases the effective axonal membrane capacitance, they reduce the charge needed to depolarize the axon and hence allow the action potential to travel much faster, by saltatory conduction from one node of Ranvier to the next, at speeds of up to 430 km/h (120 m/s) instead of ∼3.6 km/h (1 m/s) for a typical (smaller) unmyelinated axon.

During development oligodendrocytes are generated from precursor cells with a morphology that is bipolar (when migrating) or stellate (after migration). These initially differentiate into immature cells that put out processes seeking axons to myelinate, and eventually form mature cells with parallel processes myelinating up to 30 different axons. The production of myelinated axons requires a precise matching of the number of oligodendrocytes generated to the length of axons to be myelinated. This may be regulated in part by neurotransmitter receptors activated by substances released by active axons. Such interactions may also be important for maintaining the myelination of mature axons. Thus, neurotransmitter receptors play an important role in the normal life of oligodendrocytes.

Furthermore, if oligodendrocytes become damaged and the myelin sheath is destroyed, the action potential is reduced in velocity or ceases altogether, leading to physical or mental disability. This occurs in cerebral palsy (which affects about one in 500 live births), spinal cord injury (which happens to one in 1200 people), stroke (suffered by one in 450 people each year) and multiple sclerosis (present in one in 700 people). Recent work has shown that neurotransmitter receptors play a key role in damaging oligodendrocytes in pathological conditions. Understanding how this damage happens would provide some prospect of either preventing or reversing it.

In this review we will describe the neurotransmitter receptors present on oligodendrocytes, discuss how they may control oligodendrocyte development, examine how activation of these receptors contributes to oligodendrocyte damage in pathological conditions, and assess what is known about how mutations in neurotransmitter receptors or their interacting proteins may alter oligodendrocyte function.

## Neurotransmitter receptor expression in oligodendrocytes

### AMPA/kainate receptors

Glutamate evokes a membrane current in oligodendrocytes which has been reported to be generated entirely by AMPA or kainate receptors, both in culture (Barres et al., 1990; Patneau et al., 1994; Borges et al., 1994; Borges and Kettenmann, 1995; Gallo et al., 1996; Yuan et al., 1998) and in brain slices (Berger et al., 1992b). Consistent with the presence of functional AMPA and kainate receptors, oligodendrocytes express mRNA for the AMPA receptor subunits GluR2, 3 and 4, but not for GluR1 (Patneau et al., 1994; Itoh et al., 2002; Yoshioka et al., 1996; Garcia-Barcina and Matute, 1998; Matute et al., 1997; Jensen and Chiu, 1993), and for the kainate receptor subunits GluR6 and 7 and KA-1 and 2, but not for GluR5 (Jensen and Chiu, 1993; Patneau et al., 1994; Yoshioka et al., 1996; Garcia-Barcina and Matute, 1996; Matute et al., 1997). Interestingly, when detecting AMPA/kainate subunits at the protein level with immunohistochemistry, no GluR2 subunit was found in the oligodendrocytes, but GluR3 and GluR4 were expressed (Li and Stys, 2000), and immunoprecipitation experiments reported that GluR2 does not assemble with the other subunits (Puchalski et al., 1994). These data suggest that the AMPA receptors present may lack GluR2 and therefore be Ca^2+^-permeable, which has relevance for the damaging actions of glutamate on oligodendrocytes in pathology, as discussed below.

Changes in glutamate receptor expression with development may be relevant for understanding the susceptibility of oligodendrocyte precursors and immature cells to anoxia or ischemia, as discussed below, but there is profound disagreement in the literature over how the glutamate-evoked current changes with oligodendrocyte maturation. On the one hand, it has been claimed that, in culture, the glutamate response is larger in precursor or immature cells than in mature oligodendrocytes (Borges et al., 1994; Itoh et al., 2002; Deng et al., 2003; Rosenberg et al., 2003). Alternatively, in brain slices, the glutamate-evoked current has been suggested not to differ significantly between precursor oligodendrocytes and mature oligodendrocytes (Berger et al., 1992b). Finally, it has been reported that, in culture and in slices, the glutamate response in mature cells is larger than in precursors (Patneau et al., 1994; Káradóttir et al., 2005), although the increase of the glutamate-evoked current could just reflect the increase of the cell’s size with development, with no change of receptor density.

### *N*-methyl-d-aspartate (NMDA) receptors

Neuronal NMDA receptors are important for learning and memory, and for causing neuronal death in pathological conditions, but until recently were assumed to be absent from glia: for over 10 years it has been the dogma that oligodendrocytes do not express NMDA receptors. No message was detected for NMDA receptor subunits in the optic nerve (Matute et al., 1997), and NMDA was reported to generate no current in oligodendrocytes, either in culture (Barres et al., 1990; Patneau et al., 1994; Pende et al., 1994) or in brain slices (Berger et al., 1992b). However, there were some indications that NMDA receptors might be present. Yoshioka et al. (1996) reported that an oligodendrocyte precursor cell line expressed mRNA for NR1 and NR2D, and Wang et al. (1996) showed that, in culture, O-2A cells (precursor cells that can become either oligodendrocytes or type 2 astrocytes) exhibited NMDA-evoked currents that were magnesium-blocked and reduced with an NMDA antagonist. The latter finding was supported by a demonstration that mature spinal cord gray matter oligodendrocytes showed NMDA-evoked currents, but contradicted by the fact that no NMDA response was seen in the precursor oligodendrocytes which one would expect to behave like the O-2A cells studied in culture (Žiak et al., 1998); essentially no NMDA responses were detected in white matter oligodendrocytes in that study.

Recently we have shown (Káradóttir et al., 2005) that white matter oligodendrocytes express functional NMDA receptors, *in situ* in slices of cerebellum and corpus callosum, at all developmental stages, i.e. in cells expressing markers for oligodendrocyte precursors (NG2), immature cells (O4) and mature myelinating oligodendrocytes (myelin basic protein). These receptors are unusual in showing very weak block by extracellular Mg^2+^: 2 mM Mg^2+^ reduced the current only three- to fivefold relative to that seen in the absence of Mg^2+^, whereas for neuronal receptors the reduction is normally 20–70-fold. Thus, oligodendrocyte NMDA receptors generate a significant current even at the cell’s resting potential of around −60 mV. The weak Mg^2+^-block, together with antibody labeling for particular NMDA receptor subunits, suggested that the receptors might be composed of NR1, NR2C and NR3 subunits (Káradóttir et al., 2005). NR2C and NR3 subunits tend to confer a weak Mg^2+^-block on NMDA receptors (Kuner and Schoepfer, 1996; Sasaki et al., 2002), although this suggested subunit combination has not been studied in a heterologous expression system yet. Similar immunocytochemistry showed that NMDA receptor subunits are also present in optic nerve oligodendrocytes (Salter and Fern, 2005; Micu et al., 2006), suggesting that NMDA receptor expression may be a general property of oligodendrocytes throughout the brain. Although NMDA-evoked currents were demonstrated by Káradóttir et al. (2005) only over the age range investigated, from P7–P14, they demonstrated that adult rat oligodendrocytes generating compact myelin also express NMDA receptor subunits (see below).

### The subcellular localization of glutamate receptors in oligodendrocytes

**Fig. 1:**
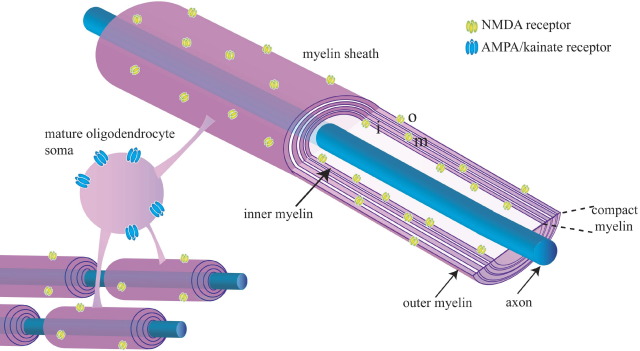
Spatial segregation of NMDA and AMPA/kainate receptors in myelinating oligodendrocytes. AMPA/kainate receptors (blue) are preferentially located on the soma, while NMDA receptors (yellow) are preferentially located on the myelinating processes, although this segregation is not absolute. Cutaway of myelin shows that NMDA receptors are found in the outer myelin wrap (o), the inner wrap (i) nearest the axon, and also deep in the compact myelin (m). This schematic diagram of the myelin ignores the fact that the oligodendrocyte cytoplasm is, in reality, thicker in the innermost and outermost turns of the myelin.

Immunocytochemical localization of NMDA receptors in mature oligodendrocytes, both in cerebellar and corpus callosum slices and in optic nerve, shows a high level of expression in the cell processes which invest axons with myelin (Káradóttir et al., 2005; Salter and Fern, 2005; Micu et al., 2006) whereas, by contrast, AMPA/kainate receptors tended to be expressed mainly in the cell soma. At the electron microscopic level, NR1 subunits were found to be present throughout the myelin, even in the compact myelin around adult axons (Káradóttir et al., 2005). The preferential location of NMDA receptors in myelinating processes (Fig. 1) suggests a possible role in controlling axon–oligodendrocyte interactions during myelination, and is consistent with activation of these receptors damaging the myelinating processes specifically in pathological conditions as discussed below. It is hard to see how receptors deep within the myelin will ever be exposed to glutamate, and they may be left there as a consequence of development. However, receptors in the inner myelin lamina should be able to respond to glutamate released from the axon being myelinated, and receptors in the outer lamina may be used for sensing glutamate release from cells surrounding the axon/myelin unit.

### Why were oligodendrocyte NMDA receptors overlooked for so long?

Several factors probably account for the long-held dogma that oligodendrocytes do not express NMDA receptors. First, the early papers reporting no NMDA-evoked currents in oligodendrocytes (Barres et al., 1990; Patneau et al., 1994; Pende et al., 1994) were on cultured cells, maintained in conditions which may down-regulate NMDA receptor expression. Second, the brain slice experiments of Berger et al. (1992b) were on corpus callosal oligodendrocytes, the NMDA receptors of which show more desensitization than for cerebellar oligodendrocytes (Bakiri et al., 2006) which may have led to an NMDA response being overlooked if NMDA was only applied after glutamate had already activated (and desensitized) the NMDA receptors. Third, the preferential location of NMDA receptors in cell processes, rather than in the cell somata where more AMPA/kainate receptors are located, may mean that in studies of glutamate damaging oligodendrocytes (which usually involve using an agent like propidium to label nuclear DNA when the cell membrane ruptures) a damaging effect of AMPA/kainate receptors may be more easily detected (see discussion below). Finally, in many studies studying the role of AMPA/kainate receptors in damaging oligodendrocytes in simulated pathology, because of the dogma that oligodendrocytes do not express NMDA receptors, and a desire to avoid NMDA receptor-mediated effects occurring in the gray matter, the “control” condition often included an NMDA receptor blocker in the solution. The outcome in this “control” condition would then be compared with what occurred when an AMPA/kainate blocker was superimposed on the NMDA blocker. This procedure automatically excludes detection of any influence of NMDA receptors.

### Metabotropic glutamate receptors (mGluRs)

mGluRs are expressed in oligodendrocytes (Deng et al., 2004), but the mGluR agonist ACPD evokes neither a current nor a rise in intracellular calcium level in oligodendrocytes (Berger et al., 1992b; Borges et al., 1994; Pende et al., 1994; Káradóttir et al., 2005), but see Holtzclaw et al. (1995) for an mGluR-mediated [Ca^2+^]_i_ rise in cultured oligodendrocyte precursors.

### Sources of glutamate to activate oligodendrocyte glutamate receptors

Oligodendrocyte precursors in both the gray and the white matter receive glutamatergic synaptic input evoked by action potentials in axons: synaptic events in oligodendrocyte precursors are blocked by TTX and by the Ca^2+^ channel blocker cadmium (Bergles et al., 2000; Káradóttir et al., 2005). Glutamate may also be released onto mature oligodendrocytes. Both frog sciatic nerve (Wheeler et al., 1966) and frog optic nerve axons (Weinreich and Hammerschlag, 1975) release preloaded radioactive glutamate in response to repetitive electrical stimulation. Moreover, axonal stimulation, or application of veratridine which generates action potentials, causes the calcium concentration to rise in mammalian optic nerve glial cells, and this is blocked by kynurenic acid (a non-specific glutamate receptor antagonist) but also by blocking glutamate transporters with dihydrokainate (Kriegler and Chiu, 1993; Chiu and Kriegler, 1994). The effect of the transporter blocker can be explained by the fact that axonal action potentials will increase the intracellular sodium concentration and the extracellular potassium concentration. This could make axonal glutamate transporters reverse their operation and release glutamate, since uptake by glutamate transporters depends on there being a low intracellular sodium concentration and a low extracellular potassium concentration: when these concentrations rise, the transporters tend to release glutamate (Szatkowski et al., 1990). In ischemic conditions, as discussed below, glutamate is released both from axons and from oligodendrocytes by reversal of glutamate transporters (Li et al., 1999; Fern and Möller, 2000). In subsequent sections we will describe the likely functional consequences of glutamate release activating its receptors on oligodendrocytes.

### GABA and other neurotransmitter receptor expression

GABA evokes a GABA_A_ receptor-mediated current in oligodendrocytes throughout their development (Pastor et al., 1995; Matute et al., 1997; Williamson et al., 1998). GABA depolarizes both mature and precursor cells (Butt and Tutton, 1992; Lin and Bergles, 2004) and raises the intracellular calcium concentration (Belachew et al., 1998; Bernstein et al., 1996; Schmidt et al., 2000; Kirchhoff and Kettenmann, 1992), suggesting that the chloride reversal potential, E_Cl_, is above the resting potential. The elevated intracellular [Cl^−^]_i_ needed to generate this depolarized value of E_Cl_ is presumably produced by the expression of NKCC1 transporters which carry Cl^−^ into the cell (Plotkin et al., 1997). Examining the message for GABA_A_ receptor subunits in precursor oligodendrocytes showed that they express mRNA for α_2_, α_3_, α_4_, α_5_, γ_2_, γ_3_ and to a lesser extent γ_1_, subunits, while δ subunits were not detected and β subunits were not tested for (Williamson et al., 1998), so the exact subunit composition of the receptors remains to be determined. Oligodendrocyte precursors in both gray and white matter receive GABAergic synaptic input from axons (Lin and Bergles, 2004; Káradóttir and Attwell, 2006), and it is conceivable that mature cells are exposed to GABA released by reversed uptake in inhibitory axons, as discussed above for glutamate.

Glycine evokes an inward current in oligodendrocyte precursor cells, mediated by strychnine-sensitive receptors gating a Cl^−^ conductance and by activation of Na^+^-dependent glycine uptake, which depolarizes the cells and increases the intracellular calcium level (Pastor et al., 1995; Belachew et al., 1998, 2000). Serotonin and ACh are reported to generate an inward current in oligodendrocytes in culture but not in slices (Belachew et al., 1998; Káradóttir et al., 2005). Histamine, norepinephrine, serotonin, angiotensin II, bradykinin, substance P, acetylcholine and ATP all raise [Ca^2+^]_i_ in oligodendrocyte precursor cells (Bernstein et al., 1996; Belachew et al., 1998; Ragheb et al., 2001). Adenosine also increases the intracellular calcium concentration, oligodendrocytes express mRNA for the A1, A2a, A2b and A3 subunits of adenosine receptors (Stevens et al., 2002), and the A1 receptor subunit has been detected at the protein level (Othman et al., 2003). Finally, oligodendrocytes at all maturational stages express mRNA for D3 dopamine receptors, so dopamine presumably lowers the cyclic AMP level in these cells (Bongarzone et al., 1998).

Below we will consider the functional significance of these receptors being present.

## The role of neurotransmitter receptors in the development of oligodendrocytes

### Axonal activity affects oligodendrocyte development

The number of mature myelinating oligodendrocytes produced during CNS development needs to be matched to the number and length of the axons requiring myelination. In addition, oligodendrocytes migrate along axons to reach their targets (Kakita et al., 2003; Small et al., 1987; Baulac et al., 1987; Kiernan and ffrench-Constant, 1993). One way to regulate these processes would be for factors to be released locally from axons to control the proliferation, differentiation and migration of oligodendrocytes. Indeed, the electrical activity of axons promotes oligodendrocyte proliferation and survival (Barres and Raff, 1993; Barres et al., 1993). If axonal electrical activity is inhibited with TTX, it disrupts oligodendrocyte development and myelination without affecting axons (Friedman and Shatz, 1990; Demerens et al., 1996) [but see, however, Colello et al. (1995) who claimed that myelination was unaffected by electrical activity in axons, and suggested that the precursors already present at the age (P0) when they blocked electrical activity were sufficient to myelinate the axons present]. In addition, an increase in axonal firing promotes the differentiation of oligodendrocytes and increases myelination (Demerens et al., 1996; Stevens et al., 2002).

Although PDGF may be one substance released, as a result of activity in axons, that influences oligodendrocyte development (Barres and Raff, 1993), there is increasing evidence for a role of smaller transmitter molecules, particularly glutamate, adenosine and ATP. As noted above, there is exocytotic release of glutamate onto oligodendrocyte precursors (Bergles et al., 2000; Káradóttir et al., 2005), and release by reversal of glutamate transporters even at later developmental stages (Li et al., 1999; Fern and Möller, 2000). Interestingly, in neonatal optic nerve, axonal stimulation increases the extracellular potassium level (from 5 to 20 mM for 20 Hz stimulation) more than in mature optic nerve (from 5 to 11 mM for 400 Hz stimulation), and the increase in potassium concentration produced by a single stimulus was seven times higher in neonatal than in mature optic nerve (Connors et al., 1982). This larger increase of [K^+^]_o_, which will promote a stronger reversal of glutamate transporters, occurs within the first postnatal week, before myelination has begun (Foster et al., 1982), suggesting that the axonal physiology (and perhaps lack of K^+^ buffering by astrocytes) facilitates more glutamate release from active axons by reversed uptake at the time in development when oligodendrocytes start to become mature.

### Effects of glutamate on oligodendrocyte proliferation and differentiation

**Fig. 2:**
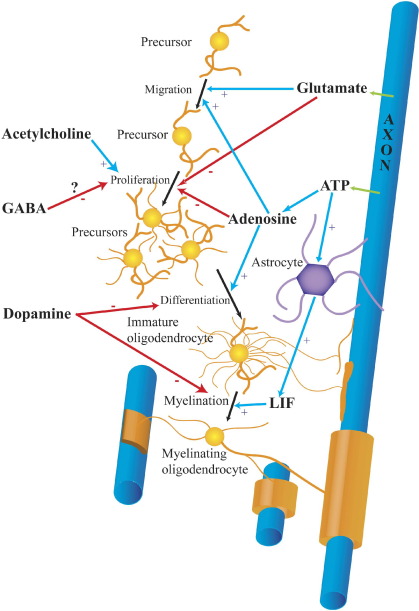
Control of oligodendrocyte development by neurotransmitters. Black arrows show the progression of oligodendrocytes from precursors, which migrate and proliferate, through immature oligodendrocytes, which send out processes seeking axons to myelinate, to mature myelinating oligodendrocytes that form myelin sheaths. Blue arrows show positive effects of neurotransmitters; red arrows show inhibitory effects. Glutamate and ATP are released (green) from active axons. Glutamate is released by exocytosis onto oligodendrocyte precursors, and by reversed uptake onto mature cells. The mechanism of release of ATP is uncertain. ATP is converted to adenosine by extracellular ATPases. ATP also induces astrocytes to release leukemia inhibitory factor (LIF). GABA is also released by exocytosis onto precursors; the origins and release mechanisms for ACh and dopamine are unknown.

Several studies suggest a role for glutamate receptors in controlling oligodendrocyte development (Fig. 2), although their results are not all mutually consistent. Firstly, activation of NMDA receptors on cultured oligodendrocyte precursor cells increased their migration rate and inhibiting their NMDA receptors decreased migration by 90%, while inhibiting AMPA receptors reduced migration by 40% (Wang et al., 1996). Gudz et al. (2006) found that the promoting effect of AMPA receptor activation on migration was associated with the formation of an α_V_ integrin/proteolipid protein/AMPA receptor complex, possibly mediated by a [Ca^+^]_i_ rise produced by the receptors, but (contradicting the work of Wang et al., 1996) they found no effect of NMDA receptor activation on migration. Secondly, glutamate acts through AMPA or kainate (but not NMDA) receptors on oligodendrocyte precursors to decrease proliferation, and also inhibits the proliferation and lineage progression induced by PDGF and bFGF (Gallo et al., 1996; Yuan et al., 1998). Even though glutamate blocked proliferation, it did not seem to promote migration (contradicting the work of Wang et al. (1996) and Gudz et al. (2006)). Interestingly, there is an interaction between glutamate and the growth factors that control proliferation. bFGF increases the expression of kainate (GluR7, KA1 and KA2) and AMPA (GluR4) receptor subunits, while PDGF increases GluR1 subunit expression, and both growth factors together increase even further the expression of GluR1 and augment the AMPA-evoked current in oligodendrocyte precursors (Gallo et al., 1994; Chew et al., 1997).

Glutamate-evoked inhibition of proliferation in oligodendrocyte precursors may serve to decrease the number of oligodendrocytes produced once the migrating precursors arrive near axons releasing glutamate. It is perhaps surprising that the growth factors that promote proliferation also increase the expression of glutamate receptors which *decrease* proliferation. However, to understand the possible roles of PDGF and glutamate it is necessary to know how far these messengers spread; conceivably PDGF spreads farther than glutamate (which is efficiently taken up) and acts on oligodendrocytes at some distance from active axons, increasing glutamate receptor expression so that proliferation is only turned off when the cells arrive near the axons and can sense the glutamate they are releasing.

The mechanism by which glutamate blocks oligodendrocyte proliferation, both in culture and in brain slices, is apparently by raising the intracellular sodium concentration, and thus inhibiting delayed rectifier K^+^ channels which are expressed in oligodendrocyte precursors but not in mature cells (Berger et al., 1991; Borges et al., 1994; Borges and Kettenmann, 1995; Gallo et al., 1996; Knutson et al., 1997; Yuan et al., 1998). The resulting inhibition of outward K^+^ flux leads to a depolarization of the cells. Consistent with a role for K^+^ channels in regulating proliferation, increased expression of the voltage-gated channel subunits Kv1.3 and Kv1.4 increases oligodendrocyte proliferation (Vautier et al., 2004), and PDGF (which also increases proliferation: Barres and Raff, 1993) increases expression of outward rectifying potassium channels (Chittajallu et al., 2005). Moreover, the glutamate-evoked inhibition of proliferation is mimicked by tetraethylammonium ions, which block K^+^ channels, and is unaffected by calcium removal (Gallo et al., 1996; Knutson et al., 1997; Yuan et al., 1998) which rules out the possibility that the inhibition of proliferation is produced by oligodendrocyte depolarization leading to activation of voltage-gated Ca^2+^ channels and a Ca^2+^ influx. The dependence of glutamate’s actions on Na^+^ entry is demonstrated by the fact that when applying glutamate (or kainate) with N-methyl-d-glucamine replacing extracellular Na^+^, the glutamate-evoked inhibition of K^+^ outward current does not occur (Knutson et al., 1997). In addition, if the intracellular Na^+^ concentration is increased with veratridine, a similar inhibition of the outward potassium current and of oligodendrocyte proliferation is produced, even in the presence of PDGF and bFGF (Knutson et al., 1997; Yuan et al., 1998).

Apart from effects of glutamate mediated by K^+^ channel closure, when glutamate activates its receptors it will also increase the intracellular calcium concentration which may activate various enzymes. Activation of non-NMDA receptors on oligodendrocyte precursor cells leads to an increase in intracellular calcium concentration, that induces an increase in gene expression since it increases mRNA levels for nuclear messengers like immediate early genes (Pende et al., 1994; Holtzclaw et al., 1995). High intracellular [Ca^2+^] could also activate protein kinase C, which inhibits differentiation and myelin basic protein expression (Baron et al., 1998).

Although the longstanding notion that oligodendrocytes do not express NMDA receptors led to a focus on developmental effects mediated by non-NMDA receptors, it is likely that the recent discovery of NMDA receptors in these cells will stimulate a reexamination of the possible role of NMDA receptor-mediated depolarization and [Ca^2+^]_i_ rise on oligodendrocyte development. In particular, the location of NMDA receptors in oligodendrocyte myelinating processes (see above) suggests a possible role for these receptors in controlling myelination.

### Effects of ATP and adenosine on oligodendrocyte development

Whereas glutamate has so far only been shown to regulate the early development of oligodendrocytes, adenosine and ATP are modulators of late oligodendrocyte development and myelination (Fig. 2). ATP is released by electrical stimulation of dorsal root ganglion axons, while adenosine may also be released or may be generated by extracellular ATPases from released ATP. Adenosine activates receptors on oligodendrocyte precursor cells, raising [Ca^2+^]_i_ and decreasing proliferation of the cells, and stimulating migration, differentiation and thus myelin formation (Stevens et al., 2002; Othman et al., 2003). ATP acts at a later stage of development, when adenosine no longer promotes myelination. It acts indirectly, inducing astrocytes to release the cytokine LIF which enhances myelination by maturing oligodendrocytes (Ishibashi et al., 2006). By contrast, in the peripheral nervous system ATP released from axons inhibits myelination by Schwann cells (Stevens and Fields, 2000).

### Effects of other neurotransmitters on oligodendrocyte development

There is only fragmentary evidence for a role of other neurotransmitters in modulating oligodendrocyte development, as follows. When GABA activates GABA_A_ receptors in oligodendrocyte precursor cells (Lin and Bergles, 2004), it may also inhibit outward rectifying potassium channels (Pastor et al., 1995), which could lead to depolarization and a reduction of proliferation as shown for glutamate (see above) and as also occurs for neurons (LoTurco et al., 1995). The acetylcholine muscarinic M3 receptor is expressed in oligodendrocyte precursors and when activated it increases proliferation by activating the MAP kinase signaling pathway (Ragheb et al., 2001). Furthermore, activation of dopaminergic D3 receptors, which are expressed in precursor and immature oligodendrocytes (but not in mature cells), decreases differentiation of the precursor cells to become mature cells and inhibits myelin formation (Bongarzone et al., 1998). Finally, since histamine, norepinephrine, serotonin, angiotensin II, bradykinin, ATP and substance P have all been shown to increase the intracellular calcium concentration in oligodendrocyte precursors (Bernstein et al., 1996), these transmitters could also influence the development of the oligodendrocytes if they are released onto the cells *in vivo*.

## The role of neurotransmitter receptors in mature oligodendrocytes

Mature myelinating oligodendrocytes respond to neurotransmitters, as documented above, but the role of this signaling is completely obscure. Conceivably constant signaling from axons to the myelin surrounding them is essential for myelination to be maintained, but this remains to be tested.

## The role of neurotransmitter receptors in oligodendrocyte pathology

### Glutamate release damages oligodendrocytes in pathological conditions

**Fig. 3:**
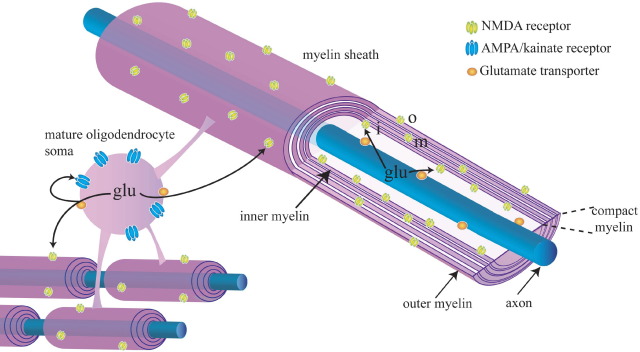
Receptor distribution defines the spatial segregation of damage expected when glutamate (glu) is released from axons and oligodendrocytes by reversal of uptake carriers (orange) in conditions of energy deprivation. Glutamate released from axons will activate NMDA receptors (yellow) on the inner wrap (i) of the myelin, leading to ion flux into the myelin and myelin damage. Glutamate released from oligodendrocytes will activate AMPA/kainate receptors (blue) on the soma (possibly leading to death of the soma) and also NMDA receptors on the inner and outer (o) wraps of the myelin.

In pathological conditions neurotransmitters can be released excessively, damaging the cells they normally act on. In the gray matter of the brain the death of neurons in pathological conditions is often caused by a rise of extracellular glutamate concentration activating NMDA receptors and causing an excessive rise of [Ca^2+^]_i_. This occurs in anoxia or ischemia (Olney, 1978; Choi et al., 1987; Choi, 1988; Arundine and Tymianski, 2004), but also in more chronic conditions such as Huntington’s or Alzheimer’s disease (Hynd et al., 2004). Glutamate can also damage white matter oligodendrocytes, in both acute and chronic diseases, including brain injury after pre- or perinatal infection, asphyxia or premature birth (periventricular leukomalacia, leading to cerebral palsy and cognitive deficits (Volpe, 2001)), spinal cord injury (Stys, 2004), multiple sclerosis (Matute et al., 2001) and stroke (Dewar et al., 2003). In perinatal asphyxia and spinal cord injury glutamate is released in the white matter by the reversal of glutamate transporters in axons and oligodendrocytes (Fig. 3) as a result of ATP depletion (Li et al., 1999; Fern and Möller, 2000), i.e. a mechanism similar to that occurring in gray matter ischemia (Rossi et al., 2000). In multiple sclerosis, the extracellular glutamate concentration may rise as a result of release by cystine–glutamate exchange in microglia/macrophages (Piani and Fontana, 1994), or because of increased glutamate production by glutaminase and reduced degradation by glutamate dehydrogenase and glutamine synthetase (Werner et al., 2001).

### Glutamate-mediated damage to oligodendrocytes mediated by AMPA/KA receptors

Until recently, there was thought to be a major difference between the excitotoxic actions of glutamate on oligodendrocytes and on neurons because, as noted above, it was thought that oligodendrocytes lacked NMDA receptors (Berger et al., 1992a; Patneau et al., 1994). Oligodendrocytes were therefore thought to be damaged solely by glutamate acting either on calcium-permeable AMPA/kainate receptors (Dewar et al., 2003; Stys, 2004; Volpe, 2001; Matute et al., 2001) or by reversing cystine–glutamate exchange and depriving the cells of antioxidant protection (Oka et al., 1993).

Consistent with a role for AMPA/kainate receptors in glutamate-mediated oligodendrocyte damage, blocking these receptors attenuates white matter injury in animal models of hypoxia/ischemia (Tekkök and Goldberg, 2001; Fern and Möller, 2000; Follett et al., 2000; Deng et al., 2003; McDonald et al., 1998), spinal cord injury (Wrathall et al., 1997; Agrawal and Fehlings, 1997) and multiple sclerosis (Smith et al., 2000; Pitt et al., 2000). In addition, white matter damage in periventricular leukomalacia occurs at a time in development when a large proportion of oligodendrocytes are precursor and immature cells (Back et al., 2002). In culture these have been reported to show a higher expression than mature cells of AMPA/kainate receptors and downstream [Ca^2+^] signaling (Itoh et al., 2002; Deng et al., 2003; Rosenberg et al., 2003), although there are reports disputing this as described above.

In addition to simple activation of AMPA/kainate receptors damaging oligodendrocytes, there is also a glutamate–immune system interaction, between the effects of activating kainate receptors and the effects of complement attack by microglia/macrophages in multiple sclerosis (or its animal model experimental autoimmune encephalomyelitis). Concentrations of glutamate which alone are not toxic, sensitize oligodendrocytes to subsequent complement attack (Alberdi et al., 2006), which inserts membrane attack complexes into the oligodendrocyte, allowing a toxic Ca^2+^ influx to occur.

### The role of NMDA receptors in glutamate-mediated damage to oligodendrocytes

Despite the long-held consensus view that glutamate damages white matter oligodendrocytes and their precursors by acting on AMPA/kainate receptors, there was some evidence that NMDA receptors might also play a role. NMDA receptor blockers slow the loss of white matter action potentials (Tekkök and Goldberg, 2001) and reduce white matter damage in ischemia (Schäbitz et al., 2000) and in an animal model of multiple sclerosis (Wallström et al., 1996).

In fact, both in oligodendrocyte precursors and in mature oligodendrocytes myelinating axons, NMDA receptors are activated by glutamate released during simulated white matter ischemia (Káradóttir et al., 2005). Activation of these receptors leads to a rise of [Ca^2+^]_i_ in the myelinating processes of mature cells (Micu et al., 2006), to which activation of AMPA/kainate receptors contributes only modestly, whereas activation of AMPA/kainate receptors is responsible for essentially all of the [Ca^2+^]_i_ rise in the cell body. This differential route of calcium entry is consistent with the segregated distribution of the different receptor classes on the cell (Fig. 3). Activation of an ion influx through the NMDA receptors on the myelinating processes leads to these processes rupturing in young animals (Salter and Fern, 2005) and in adult animals to the compact myelin becoming seriously deformed (Micu et al., 2006). A likely factor contributing to this damage to the myelinating processes is the small intracellular volume of these processes, which will result in a large fractional concentration change of [Na^+^] and [Ca^2+^]_i_ in the cell, resulting in water influx and swelling. The resulting disruption of the myelin sheath (Salter and Fern, 2005; Micu et al., 2006) must slow or abolish action potential propagation, but at present it is unknown whether oligodendrocytes can recover from this damage to their processes. In addition it is unclear whether damage to the processes mediated by NMDA receptors will be detected by propidium iodide as commonly used to detect rupture of the plasma membrane: conceivably when the processes become damaged the rest of the cell seals off its membrane and the soma can live on and perhaps regenerate new processes, providing the rise of glutamate around the soma does not activate AMPA/kainate receptors sufficiently to kill the soma.

### Oligodendrocyte NMDA receptors as a therapeutic target in white matter disease

So far a role for NMDA receptors in damaging oligodendrocytes has only been directly shown in simulated ischemic conditions. However, since the extracellular glutamate concentration is thought to rise in the white matter not only in ischemia, but also in spinal cord injury and multiple sclerosis, it is likely that a similar damage induction process occurs in these other conditions (just as activation of oligodendrocyte AMPA/kainate receptors occurs not only in ischemia but also in animal models of multiple sclerosis: Pitt et al., 2000; Smith et al., 2000). This suggests that oligodendrocyte NMDA receptors would be a useful therapeutic target in a range of white matter diseases.

The fact that these receptors have a subunit stoichiometry that differs from most neuronal NMDA receptors (see above) suggests the possibility of devising a drug that blocks these receptors specifically, avoiding the problems of side effects on neuronal receptors that have prevented useful therapeutic application of NMDA receptor blockers in stroke (Lipton, 2006). Furthermore, the damage to the white matter which occurs in spinal cord injury (as a secondary consequence of damage to blood vessels) and in multiple sclerosis progresses slowly, which should give a therapeutic time window in which NMDA blockers could be given. Interestingly memantine, one of the few NMDA antagonists licensed for use in humans, blocks the oligodendrocyte NMDA receptors (Bakiri et al., 2006), and so might provide a useful starting point for testing the therapeutic effects of NMDA blockers in white matter diseases.

### The role of GABA and other neurotransmitters in white matter ischemia

Just as glutamate receptors play a role in damaging oligodendrocytes in pathological conditions, it is possible that other neurotransmitter receptors on these cells may exacerbate or reduce damage to oligodendrocytes in white matter diseases.

In both the gray and the white matter there is an increase in the extracellular GABA concentration during ischemia (Shimada et al., 1993). In gray matter, GABA release is either thought to be beneficial in ischemia, as it will decrease the neuronal depolarization caused by glutamate, or thought to be harmful, as it can cause cell damage by inducing an influx of Cl^−^ ions through GABA_A_ receptors, followed by water influx and cell swelling (Inglefield and Schwartz-Bloom, 1998; Allen et al., 2004). In the white matter GABA_A_ receptors on oligodendrocytes, some of which are localized in the myelinating processes, are activated during simulated white matter ischemia (Káradóttir et al., unpublished observations) but the situation is slightly different, because E_Cl_ is positive to the resting potential (Lin and Bergles, 2004; Kirchhoff and Kettenmann, 1992; and see above). Thus, activating GABA_A_ receptors will depolarize the cell (perhaps evoking a Ca^2+^ influx through voltage-gated Ca^2+^ channels) but, in contrast to the situation in neurons, will let Cl^−^ out of the cell and decrease intracellular osmolarity. This activation of GABA_A_ receptors during ischemia has been reported to decrease action potential conduction in the spinal cord (Lee et al., 1993). Interestingly the anti-epileptic drug vigabatrin, which raises extracellular GABA levels by inhibiting the GABA degrading enzyme GABA transaminase, can cause swelling and loss of myelin (Sidhu et al., 1997), suggesting that excessive activation of GABA_A_ receptors in the myelinating processes of oligodendrocytes may damage those processes.

On the other hand, Fern et al. (1995) reported that GABA release ameliorated action potential conduction loss in optic nerve ischemia by acting through GABA_B_ receptors. Similarly activation of oligodendrocyte dopamine D_2_ and D_3_ receptors by exogenous agonists has been suggested to reduce oligodendrocyte damage caused by glutamate or energy deprivation (Rosin et al., 2005), and melatonin reduces oxidative damage to white matter after activation of glutamate receptors or ischemia either by inhibiting adenylate cyclase or through its free radical scavenging activity (Husson et al., 2002; Lee et al., 2005).

## Genome instability and neurotransmitter signaling to oligodendrocytes

A variety of neurological disorders are associated with mutations in neurotransmitter receptors, transporters or associated proteins. For example, in mice, the *lurcher* mutation in the GluRdelta2 subunit and the *weaver* mutation in a G protein-gated inwardly rectifying channel cause movement disorders by disrupting cerebellar function, while the *stargazer* mutation in a protein regulating trafficking of AMPA receptors and calcium channels leads to absence seizures and ataxia. In humans, GABA_A_ and nicotinic ACh receptor mutations can cause epilepsy, and neuroticism and depression are associated with a polymorphism in the promoter for the 5-HT transporter (Steinlein et al., 1995; Baulac et al., 2001; Harkins and Fox, 2002; Letts, 2005; Vogel et al., 2006; Munafo et al., 2006).

A large number of genetic disorders are also known to affect the white matter. These largely reflect myelination defects due to mutations causing defects in enzymes such as arylsulphatase A (metachromatic leukodystrophy), aspartoacylase (Canavan disease), galactosylceramidase (Krabbe disease) or enzymes breaking down phytanic acid (Refsum’s disease), defects in a fatty acid transfer protein (adrenoleukodystrophy), or defects in the myelin protein PLP1 (Pelizaeus-Merzbacher disease). Although our understanding of neurotransmitter signaling to oligodendrocytes is in its infancy, it is likely that genetic disorders of this signaling, similar to those in gray matter, will also occur, even if in some cases their effects may be masked by effects of the mutation on gray matter function. In addition, when mutations occur in oligodendrocytes in molecules not related to neurotransmitter signaling, it is possible that neurotransmitter signaling to the oligodendrocytes may modulate the consequences. Here we highlight three possible examples.

First, glutamatergic signaling to oligodendrocytes is expected to depend on glutamate transporters to maintain a low extracellular glutamate: indeed, blocking these transporters raises the extracellular glutamate concentration and damages oligodendrocytes and axons (Domercq et al., 2005). A common polymorphism in the promoter for the glutamate transporter EAAT2/GLT1, which is expressed in white matter (Domercq and Matute, 1999; Domercq et al., 1999), leads to less transporter expression, a raised extracellular glutamate concentration and worse outcome in stroke (Mallolas et al., 2006). It seems likely that this polymorphism will also predispose to worse outcome in white matter disorders that are caused in part by glutamate, such as spinal cord injury and multiple sclerosis.

Second, some cases of Alzheimer’s disease are caused by mutations in the presenilin-1 gene (Sherrington et al., 1995), which generates part of the gamma secretase complex that cleaves amyloid precursor protein, releasing the amyloid β peptide which is deposited in amyloid plaques in this disorder. Interestingly, glutamate-mediated damage to oligodendrocytes is potentiated by a presenilin-1 mutation, possibly as a result of amyloid β impairing intracellular calcium regulation so that glutamate evokes a larger [Ca^2+^]_i_ rise in the cells (Pak et al., 2003).

Finally, neurotransmitter signaling may modulate the consequences of mutations causing oligodendrogliomas, a significant class of brain cancer. Glioma malignancy correlates inversely with GABA_A_ receptor expression (Jussofie et al., 1994). Since GABA and glutamate suppress the proliferation of neurons (LoTurco et al., 1995) and possibly oligodendrocytes (see above and Fig. 2), and oligodendroglioma cells (like oligodendrocytes) express GABA_A_ and glutamate receptors (Labrakakis et al., 1998a, b), it is possible that GABAergic and glutamatergic signaling could inhibit oligodendroglioma growth.

## Conclusions

In comparison with the gray matter, neurotransmitter signaling in the white matter has received relatively little attention, but it is likely to play a major role in both the life and death of oligodendrocytes. Novel therapeutic strategies for treating white matter diseases may be possible based on the presence in oligodendrocytes of NMDA receptors with a subunit stoichiometry that differs from that of most NMDA receptors in neurons.
